# Feasibility and long-term efficacy of a proactive health program in the treatment of chronic back pain: a randomized controlled trial

**DOI:** 10.1186/s12913-019-4561-8

**Published:** 2019-10-21

**Authors:** A. Hüppe, C. Zeuner, S. Karstens, M. Hochheim, M. Wunderlich, H. Raspe

**Affiliations:** 1 Institute of Social Medicine and Epidemiology, University of Lübeck, Ratzeburger Allee 160, 23562 Lübeck, Germany; 2 Department of Computer Science, Therapeutic Science, Trier University of applied Science, Schneidershof, 54293 Trier, Germany; 3 Generali Health Solutions GmbH, Hansaring 40-50, 50670 Köln, Germany; 4 Central Krankenversicherung AG, Strategisches Leistungs- und Gesundheitsmanagement, Hansaring 40-50, 50670 Köln, Germany; 5 Institute for Ethics, History and Theory of Medicine , University of Münster, von Esmarch-Straße 62, 48149 Münster, Germany

**Keywords:** Back pain, Health services research, Evaluation, Patient-reported outcomes

## Abstract

**Background:**

To facilitate access to evidence-based care for back pain, a German private medical insurance offered a health program proactively to their members. Feasibility and long-term efficacy of this approach were evaluated.

**Methods:**

Using Zelen’s design, adult members of the health insurance with chronic back pain according to billing data were randomized to the intervention (IG) or the control group (CG). Participants allocated to the IG were invited to participate in the comprehensive health program comprising medical exercise therapy and life style coaching, and those allocated to the CG to a longitudinal back pain survey. Primary outcomes were back pain severity (Korff’s Chronic Pain Grade Questionnaire) as well as health-related quality of life (SF-12) assessed by identical online questionnaires at baseline and 2-year follow-up in both study arms. In addition to analyses of covariance, a subgroup analysis explored the heterogeneity of treatment effects among different risks of back pain chronification (STarT Back Tool).

**Results:**

Out of 3462 persons selected, randomized and thereafter contacted, 552 agreed to participate. At the 24-month follow-up, data on 189 of 258 (73.3%) of the IG were available, in the CG on 255 of 294 (86.7%). Significant, small beneficial effects were seen in primary outcomes: Compared to the CG, the IG reported less disability (1.6 vs 2.0; *p* = 0.025; d = 0.24) and scored better at the SF-12 physical health scale (43.3 vs 41.0; *p* < 0.007; d = 0.26). No effect was seen in back pain intensity and in the SF-12 mental health scale. Persons with medium or high risk of back pain chronification at baseline responded better to the health program in all primary outcomes than the subgroup with low risk at baseline.

**Conclusions:**

After 2 years, the proactive health program resulted in small positive long-term improvements. Using risk screening prior to inclusion in the health program might increase the percentage of participants deriving benefits from it.

**Trial registration:**

The trial was registered at the German Clinical Trials Register under DRKS00015463 retrospectively (dated 4 Sept 2018).

## Background

Back pain (BP) in Germany, as is the case worldwide, is a health disorder of high epidemiological, medical and economic importance [1–3]. Since years, they have been causing high direct and indirect costs, as they are a particularly frequent reason for the use of the medical care system, incapacity for work, and for claiming disability pension [4, 5]. National and international guidelines for evidence-based diagnosis and treatment of acute and chronic BP are available; their recommendations cover important aspects of care and are mostly consistent with each other [6–9]. However, successful implementation of guideline recommendations is hampered by various barriers [10], and in practice there is continued overuse and misuse [11]. For example, although in the media there has been intensive dissemination of the message that staying physically active is important for relief of BP, every second participant in a representative survey considers “resting the back” to be an effective means of alleviating complaints [12]. In particular, doctors with a strong biomedical understanding of disease prescribe rest and bed rest and tend not to follow treatment guidelines [13].

The German health care system is characterized by free choice of doctor and the obligation to insure all citizens. If the annual income exceeds a certain limit, one can freely choose between a statutory and a private health insurance (dual system of health insurance); about 11% of Germans are privately insured. Case management of BP is a challenge for both statutory and private German health insurances. All health insurances would like to ensure that their policyholders are given evidence-based care that avoids overuse, underuse or misuse. Whereas for some chronic diseases (e.g. diabetes mellitus), uniform, guideline-based, structured treatment programs (DMP = disease management program) have already been developed, these are still lacking for BP. The legal basis for such a DMP for BP is currently being prepared [14]. Till now, insured persons with a high illness burden (such as many days of incapacity to work due to BP) have been offered various back health programs in different ways by case managers of their respective health insurance companies. Such an approach has seldom been accompanied by scientific research [15, 16].

In order to facilitate timely access to evidence-based care for insured individuals with chronic BP, one of the 10 largest German private health insurers designed a health service designated “initiative.back”. It includes treatment by an interdisciplinary network of therapists, and individual coaching by phone is offered in parallel with the tailored treatment path. This private health insurance provider, acting proactively, invited in writing those of its members whose billing data suggested that they suffered from chronic BP to participate in the treatment program.

An evaluation study was carried out in parallel with the implementation of the treatment program. Besides feasibility and acceptance, efficacy, benefit and cost analyses were additional objectives of this study. First follow-up data collected shortly after the end of the program gave reason to suppose that this approach had beneficial effects [17]. In this study, we analyze the long-term effects on outcomes as reported by patients and discuss ways to improve the effectiveness of this approach to treatment of BP. Cost analyses are still pending and will be published separately.

## Methods

### Study design and recruitment

The study was conducted as a parallel group randomized controlled trial using Zelen’s design. The specific characteristic of Zelen’s design (also called randomized consent design) is that consent to participate is sought only after randomization [18, 19]. The study adheres to CONSORT guidelines.

Eligible participants were members of the German Private Health Insurance Central with a minimum age of 18 years and showing symptoms and “administrative signs” of chronic BP. They were selected by the employees of the health insurance company on the basis of predefined selection criteria (see Table 1) and analysis of existing billing data on the insured. The selection criteria were chosen in such a way that the identified persons were highly likely to suffer from chronic BP. The billing data used for this included treatment and cost information on outpatient treatment (e.g. drugs), inpatient treatment (e.g. surgeries) and daily sickness allowance. Conclusions about the disease were drawn from invoices submitted by the insured persons which also included the ICD codes based on which treatment choices had been made [20, 21].

**Table 1:** Search keys to identify potential study participants based on health insurance billing data

Inclusion criteria	- at least 18 years - at least 2 cases assigned to ICD codes M40-M54 (dorsopathies) - at least one of the following three: • one or more cases of temporary work disability in the past 12 months assigned to ICD codes M40-M54 or • two or more opioid prescriptions or • one or more cases from the following list of ICD-10 codes: F32 depressive episode; F33 recurrent depressive disorder; F34 persistent mood [affective] disorders; F38 other mood [affective] disorders; F41.2 mixed anxiety and depressive disorder; F43.2 adjustment disorders; F45.4 persistent somatoform pain disorder; F48.0 neurasthenia; F54 psychological and behavioral factors associated with disorders or diseases classified elsewhere; F62.8 chronic pain personality syndrome
Exclusion criteria	- nursing care level II or III (long-term care insurance act, SGB XI) - in the past 12 months more than one billing and settlement for the same diagnosis or for 5 or more different diagnoses from the following list of ICD-10 codes: • B16.-16.9 acute hepatitis B; B17.1 acute hepatitis C; B20–24 human immunodeficiency virus; D00-D09.9 in situ neoplasms; F00-F09 organic, including symptomatic, mental disorders; F10-F19 mental and behavioral disorders due to psychoactive substance use without F17 (mental and behavioral disorders due to use of tobacco; F20-F29 schizophrenia, schizotypal and delusional disorders; F30 manic episode; F31 bipolar affective disorder; F42 obsessive-compulsive disorder; F60 specific personality disorders; G00–09 inflammatory diseases of the central nervous system; G10 Huntington disease; G13 systemic atrophies primarily affecting central nervous system in diseases classified elsewhere; G23 other degenerative diseases of basal ganglia; G30–32 other degenerative diseases of the nervous system; G37.9 demyelinating disease of central nervous system, unspecified; G92 toxic encephalopathy; G93 other disorders of brain; H54.0 blindness, binocular; H91.3 deaf mutism, not elsewhere classified; I64 stroke, not specified as hemorrhage or infarction; K74 fibrosis and cirrhosis of liver; N18 chronic kidney disease; R54 senility

Between April and October 2015, eligible members of the private health insurance were randomly allocated to the intervention (IG) or the control group (CG) by the study center at the university. Simple block randomization was conducted by an independent external researcher using BiAS for windows version 11.02. The allocation ratio was 4 to 3 to compensate for anticipated different participation rates in IG and CG. The private medical insurance invited the allocated members in writing to participate in the study arm to which they were assigned without disclosure of the “pre” randomization step (Zelen’s design). The invitation letter described the target group as persons with BP over several months. Informed consent was obtained from the IG for participation in the health program and follow-up measurements to evaluate the effects, and from the CG, for participation in a follow-up study to evaluate the effects of usual care for chronic BP. Members of IG and CG filled in identical online-questionnaires at home at baseline as well as one and 2 years thereafter. Between April and October 2017 data collection ended with the two-year follow-up.

### Intervention for IG members

The main elements of the health program “initiative.back” under evaluation were as follows:

(1) IG-members were advised to consult a physician from a network of back experts (composed of general practitioners, orthopedic specialists, pain therapists, psychotherapists, physiotherapists), all of them following the recommendations of the National Disease Management Guideline on non-specific low BP [8, 9], such as interdisciplinary assessment and multimodal treatment for patients with chronic or recurrent BP. The initial examination by the physician also included investigation of the back. Based on the examination results, a tailor-made therapy program for the back muscles, safe from a medical point of view, was put together for each participant in specialized back centers. Participants received equipment-based training for a maximum of 24 h over a period of three to 4 months. Each of these lasted 60 min and included a combination of strength training, gymnastics and relaxation exercises to strengthen the back muscles and relieve the strain on the spine (FPZ-therapy [22], for details see http://www.fpz.de).

(2) Each IG member received personal health coaching over the phone from an external professional coach (not employed at the private medical insurance). Participants were coached during the treatment phase as well as up to 6 months thereafter in the context of after-care. A maximum of 222 min spread over 16 contacts with each participant was planned, but frequency and duration of coaching over the phone were geared to individual needs. Coaching aimed at encouraging life style changes and the consolidation of physical activities. During aftercare the participants were eligible to receive twice an activity bonus of 100 Euros each if they participated in any sports activities of their choice.

The maximum duration of the total health program was 12 months.

For evaluation of acceptance, the health insurance company provided information on participation in the health program (entire program completed, participation in the program prematurely terminated or program not joined) and on the intensity of use (number of therapeutic exercise sessions, duration of coaching over the telephone).

### Usual care for CG

The CG members did not undergo any study intervention, receiving only “usual care” i.e. care according to the prescriptions of their health care providers (family doctors or medical specialists). Information on care procedures for their BP was not available. Therefore, it is not clear to what extent treatment of BP was in accordance with the recommendations of the National Clinical Practice Guideline for Non-Specific Low BP [8, 9].

### Primary and secondary patient-reported outcome measures

Severity of BP as one of the two primary outcomes was assessed by the German version of the Chronic Pain Grade Questionnaire (CPGQ) [23, 24]. The CPGQ is a brief and simple instrument to hierarchically grade the severity of chronic pain in terms of pain intensity and disability and can be used in general population-based studies as well as in those relating to pain patients in primary care. In the presented study we measured intensity of BP and BP-related disability using the recommended scoring rules [23]. Intensity was calculated as the average of three 0 to 10 ratings on current BP, worst BP and average BP (in the past 6 months) and was expressed as a percentage value of 0 to 100% (with higher scores indicating more severe pain). BP-related disability was expressed as disability points. These were determined on the basis of the number of self-reported disability days in the past 6 months (≤6 days = 0 points, 7–14 days = 1 point, 15–30 days = 2 points, ≥31 days = 3 points) and the average of three 0 to 10 ratings on experienced impairments in daily, family/social and work/household activities, expressed as a percentage value of 0 to 100% (≤ 29% = 0 points, 30–49% = 1 point, 50–69% = 2 points, ≥70% = 3 points). Disability points are the sum of points for disability days and impairments in activities and range from 0 to 6 points with higher scores indicating more severe disability. BP severity can be graded in 4 hierarchical classes: Grade I (disability points < 3, pain intensity < 50%), Grade II (disability points < 3, pain intensity > 50%), Grade III (disability points = 3–4) and Grade IV (disability points = 5–6).

Health-related quality of life (HRQoL), the other primary outcome, was assessed with the German Short Form 12 (SF-12) [25], a generic health status instrument. Physical and mental health composite scores were computed, each ranging from 0 to 100, where zero indicates the lowest health status measured and 100 the highest.

Secondary outcomes included the risk of BP chronification measured by the Keele STarT Back Screening Tool, German version (STarT-G). The STarT-G consists of nine items. The first four items relate to biomedical factors and the remaining five identify psychosocial risk factors. A total score (ranging from 0 to 9 points) and a psychosocial sub-score (ranging from 0 to 5 points) are calculated. Patients can then be allocated to one of three prognostic groups using established scoring cut-offs (low-risk: total score ≤ 3 points; medium-risk: total score > 3 and sub-score < 4 points; high-risk: total score > 3 and sub-score ≥ 4 points) [26–28].

Psychological distress was assessed with the Patient Health Questionnaire-4 (PHQ-4), a 4 item inventory rated on a 4 point Likert-type scale. It is composed of the first two items of the Generalized Anxiety Disorder–7 scale (GAD–7) and the Patient Health Questionnaire-8 (PHQ-8). PHQ-4 total score is determined by adding together the scores of each of the four items, ranges from 0 to 12, with higher scores indicating more emotional distress (anxiety and depression) [29, 30].

Physical activity was measured with two questions referring to the last 3 months: “On how many days are you physically active on average in a way that you start to sweat or get out of breath?” Active participants were further asked: “How long are you physically active on average on these days?” Possible answers were: “less than 10”, “10 to less than 30”, “30 to less than 60” or with “more than 60” min [31].. As outcome parameter we used the number of days per week with at least 10 min of physical activity a day.

### Sample size

The sample size was calculated on the ability to detect a statistically significant difference in the primary outcomes between IG and CG at the 2-year follow-up with a small effect size of Cohen’s d = 0.3, a 2-sided α = 0.05 and a test power of 1-β = 0.8. Anticipating a dropout rate of up to 40%, we aimed at having 290 participants per study arm to ensure a sample size of at least 176 participants per study arm with data at the 2-year follow-up.

### Statistical methods

Statistical analysis was performed on an intention-to-treat basis. Each participant was analyzed in the study group to which he or she was randomized. Only participants with complete data (baseline and 2-year follow-up) were analyzed. Dropout analyses were conducted to estimate attrition bias. If a question was left unanswered, the participant could not proceed further till it was filled in. The online questionnaire, thus structured, prevents single missing values.

For each study group we presented unadjusted means and standard deviations (baseline and 2-year follow up) and reported within-group differences (time effects) using *p*-values from dependent-sample t-tests. The magnitude of changes over time was estimated with Standardized Response Mean (SRM). To assess the 2-year effects of the integrated treatment concept, analyses of covariance (ANCOVAs) were conducted for primary and secondary outcomes. As covariates, we used the baseline score of the outcome variable together with other significant (α = 0.05) differences between IG and CG at baseline.

All significance tests were performed without α adjustment. Due to multiple comparisons the results have a descriptive character [32]. Effects sizes for the between-group differences were calculated as Cohen’s d (or Hedges’ g) with 95% confidence intervals [33].

In addition to the primary analyses, subgroup analyses were done to explore the heterogeneity of treatment effects in participants with different risks of BP chronification. For the primary outcomes, we contrasted treatment effects in persons with medium or high risk of BP chronification (STarT-G total score > 3) at baseline with treatment effects in persons with low risk (STarT-G total score < 3). The between-subgroup interaction test of Altman was used to assess if potential treatment differences depended on the person’s subgroup [34–37].

Statistical analyses were performed using IBM SPSS Statistics 22. For the computation of effect sizes, the free software “Psychometrica” was used [38].

### Ethical aspects, registration, funding

Written informed consent was obtained from all study participants. The independent research ethics committee of the University of Lübeck gave approval for the study (Re.-No.14–249, dated 20 Nov 2014). The procedure for collecting and processing the study data was agreed upon with the data protection officer of the private health insurance company. The contract research study was supervised by the Lübeck research group within the framework of the contract with the insurance company. The trial was registered at the German Clinical Trials Register under DRKS00015463 retrospectively (dated 4 Sept 2018).

## Results

### Participation

**Fig. 1:**
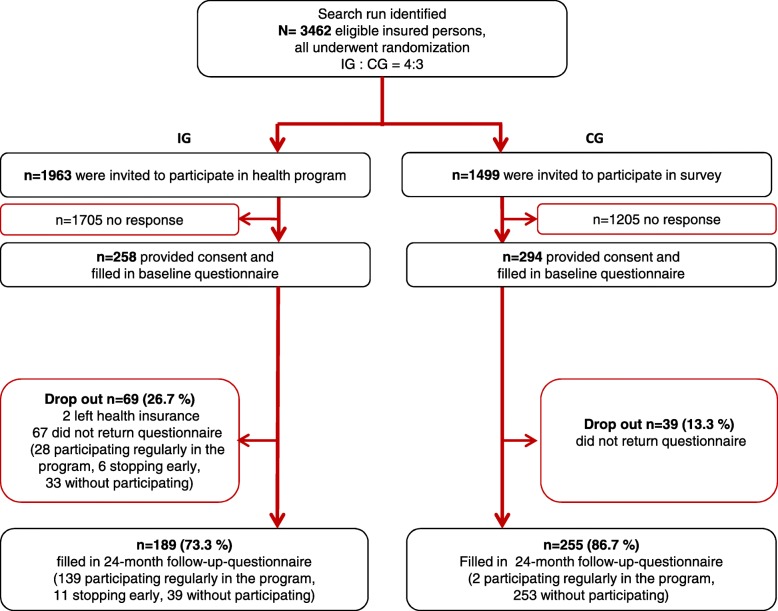
Flowchart 24-month follow-up

A total of 3462 insured persons were randomized and contacted. Of these, 552 gave their consent to participate in this study. The participation rate was significantly lower in the IG (*N* = 258, 13.1%) than in the CG (*N* = 294, 19.6%) (*p* < 0.001). The follow-up questionnaire was completed by 444 (80.4%) participants 2 years later. The IG and CG showed different dropout rates (IG: 26.7%, CG: 13.3%, *p* < 0.001) (see Fig. 1).

Table 2 shows participant characteristics at baseline.

**Table 2:** Characteristics of study participants at baseline (complete data set)

Characteristics		Total (*N* = 444)	IG (*N* = 189)	CG (*N* = 255)
		N (%)/ M (SD)	N (%)/ M (SD)	N (%)/ M (SD)
Gender	female	163 (36.7%)	60 (31.7%)	103 (40.4%)
Age	years	53.5 (8.5)	53.4 (8.1)	53.6 (8.7)
Schooling	maximum 9 years	52 (12.0%)	23 (12.5%)	29 (11.6%)
	10/11 years	183 (42.1%)	79 (42.9%)	104 (41.4%)
	12/13 years	200 (46.0%)	82 (44.6%)	118 (47.0%)
Region	urban areas	342 (77.0%)	155 (82.0%)	187 (73.3%)
	rural areas	102 (23.0%)	34 (18.0%)	68 (26.6%)
Back pain severity (CPGQ)	grade I	180 (40.6%)	64 (33.9%)	116(45.5%)
	grade II	62 (14.0%)	26 (13.8%)	36 (14.1%)
	grade III	101 (22.7%)	52 (27.5%)	49 (19.2%)
	grade IV	101 (22.7%)	47 (24.9%)	54 (21.2%)
General health (SF-12, single item)	fair/poor	180 (40.5%)	83 (43.9%)	97 (38.0%)
Psychological distress (PHQ-4)	no distress (< 3)	194 (43.7%)	79 (41.8%)	115 (45.1%)
Risk of back pain chronification (STarT-G)	low (0–3)	262 (59.0%)	100 (52.9%)	162 (63.5%)
	medium (4–7)	135 (30.4%)	64 (33.9%)	71 (27.8%)
	high (8–9)	47 (10.6%)	25 (13.2%)	22 (8.6%)
Body mass index	kg/m^2^	27.4 (4.8)	27.6 (4.8)	27.2 (4.9)
Sedentary time^a^	75% or more	212 (47.7%)	99 (52.4%)	113 (44.3%)
Sports activity (last 3 months)	no sports (yes)	96 (21.6%)	42 (22.2%)	54 (21.2%)
Satisfied with medical care of back pain (NRS)	0 = not at all content;10 = very content	6.2 (2.7)	5.7 (3.0)	6.5 (2.5)

*N* number valid cases, *M* mean, *SD* Standard deviation, *NRS* Numeric rating scale, *IG* Intervention group, *CG* Control group, *CPGQ* Chronic Pain Grade Questionnaire according to v. Korff; STarT: Screening Instrument STarT Back Tool; SF 12: 12 Items Short Form questionnaire, *PHQ-4* Patient Health Questionnaire-4; ^a^time spent sitting on a typical day

IG and CG members showed comparable sociodemographic characteristics. Significant differences were seen in severity of BP (IG worse than CG), in the risk of BP chronification (IG higher risk than CG) as well as in satisfaction with medical care of BP (IG less satisfied than CG).

### Dropout analyses

Analyses were done for study participants. Because of the different drop-out rates in IG and CG, the study groups were analyzed separately. There were few significant differences in the demographic and clinical characteristics at baseline between responders and those lost to the 24-month follow-up (non-responders). At the baseline, the non-responders in IG as well as CG differed in one of the 12 characteristics listed in Table 2. The non-responders in the IG were significantly more dissatisfied with the previous BP treatment than the responders (4.8 versus 5.7; *p* = 0.038). There were significantly more men among the non-responders than among the responders in the CG (76.9% compared to 59.6%; *p* = 0.038).

Among the responders in the IG, the proportion of study participants who completed the health program was significantly higher than among the non-responders (73.5% versus 40.6%; *p* < 0.001) (see Fig. 1).

### Acceptance of health program

Approximately one in eight of the insured persons who were invited to participate in the initiative.back accepted this offer (258 out of 1963). Among these, about 2 out of 3 (167 out of 258) completed the health program, about 7% (17 out of 258) terminated it prematurely, and 28% (72 out of 258) quit the program even before starting on it (see Fig. 1). The most frequently cited reason was the inconvenient distance from the place of residence to the nearest medical practice or training center.

Of those who participated in both the program and the 24-month follow-up, 91% underwent the maximum of 24 h of exercise therapy spread over the entire duration of therapy, 9% received only 10 h. On average, 191 min of coaching over the telephone per capita was realized (SD = 62; range 51–443 min).

### Long-term treatment effects

As far as changes over time are concerned (see Table 3), in the IG, significant improvements were observed in 6 of the 7 outcomes (excluding mental health) and in the CG, in 3 outcomes (pain intensity, disability and mental health status). All observed positive changes were in the small range (SMR < 0.5).

**Table 3:** Within-group changes in IG and CG on primary and secondary outcomes

		Baseline	24 m post	Baseline	24 m post	Within group	
		M (SD)	M (SD)	M (SD)	M (SD)	p / SRM	p / SRM
		IG (*N* = 189)		CG (*N* = 255)		IG	KG
Back pain Severity (CPGQ)	characteristic pain intensity (0–100%)	46.3 (19.1)	39.9 (21.8)	44.3 (20.4)	40.6 (22.2)	< 0.001 / 0.32	0.001 / 0.21
	disability points (0–6)	2.6 (2.1)	1.8 (2.1)	2.2 (2.2)	1.9 (2.1)	< 0.001 / 0.37	0.005 / 0.18
Health-related quality of life (SF-12)	physical component (0–100)	37.6 (9.2)	42.3 (10.1)	40.5 (11.1)	41.7 (10.8)	< 0.001 / -0.48	0.058 / –0.12
	mental component (0–100)	46.5 (12.3)	47.2 (10.3)	44.5 (12.1)	46.5 (12.2)	0.384 / –0.06	0.004 / –0.18
Psychological distress (PHQ-4)	sum score (0–12)	3.4 (2.9)	2.8 (2.7)	3.4 (2.8)	3.1 (2.6)	< 0.001 / 0.21	0.076 / 0.11
Risk of chronifi-cation (STarT-G)	total score (0–9)	3.7 (2.2)	2.8 (2.3)	3.1 (2.3)	2.9 (2.3)	< 0.001 / 0.38	0.077 / 0.11
Physical activity^a^	days per week with at least 10 min physical activity (0–7)	2.2 (1.6)	2.6 (1.6)	2.5 (1.8)	2.5 (1.7)	< 0.001 / –0.28	1.0 / 0.0

*CPGQ* Chronic Pain Grade Questionnaire, *SF-12* Short Form 12, *PHQ-4* Patient Health Questionnaire 4, *STarT-G* Keele STarT back Screening Tool, German Version;

^a^physically active in a way that one starts to sweat or gets out of breath; M: mean; SD: standard deviation; 24 m post: 24 months follow-up; IG: intervention group; CG: control group; p: significance value (from dependent sample t-test);

*SRM* Standardized response mean, minus SRM: if higher scores of the outcome parameter describe worse health condition (e.g. pain intensity or disability points), minus SMR indicates deterioration over time; if higher scores describe better health conditions (e.g. SF-12 parameters), minus SRM indicates improvement over time

To assess the long-term treatment effects, we compared the outcome variables between IG and CG at the 2-year follow-up adjusted for baseline differences. In 5 of 7 outcomes, the IG reached significantly more favorable scores than the CG (see Table 4).

**Table 4:** Between-group comparisons on primary and secondary outcomes at 24-month follow-up (ANCOVA)

		IG (*N* = 189) M^+^ (95% CI)	CG (*N* = 255) M^+^ (95% CI)	Between group	
				p	Cohen’s d* [95% CI]
*Primary outcomes*					
Back pain severity (CPGQ)	characteristic pain intensity (0–100)	38.7 (36.2–41.2)	41.5 (39.3–43.6)	0.110	−0.16 [− 0.34 to 0.03]
	disability points (0–6)	1.6 (1.4–1.8)	2.0 (1.8–2.2)	0.025	−0.24 [− 0.43 to − 0.05]
Health-related quality of life (SF-12)	physical component (0–100)	43.3 (42.0–44.5)	41.0 (39.9–42.0)	0.007	0.26 [0.07 to 0.45]
	mental component (0–100)	46.9 (45.5–48.3)	46.7 (45.5–48.0)	0.855	0.02 [−0.21 to 0.17]
*Secondary outcomes*					
Psychological distress (PHQ-4)	sum score (0–12)	2.7 (2.4–3.1)	3.2 (2.9–3.4)	0.040	−0.24 [− 0.42 to − 0.05]
Risk of chronifi-cation (STarT-G)	total score (0–9)	2.6 (2.3–2.8)	3.0 (2.8–3.3)	0.009	− 0.25 [− 0.44 to − 0.07]
Physical activity^a^	days per week with at least 10 min physical activity (0–7)	2.7 (2.5–2.9)	2.4 (2.2–2.6)	0.028	0.21 [0.03 to 0.40]

*IG* intervention group, *CG* Control group, *p* significance value; M^+^: values of the group means adjusted for baseline-score, StarT-G total risk score and satisfaction with medical care of back pain at baseline, *CI* Confidence interval; CPGQ Chronic Pain Grade Questionnaire; SF-12: Short Form 12; PHQ-4: Patient Health Questionnaire 4; STarT-G: Keele STarT back Screening Tool, German Version

^a^physically active in a way that one starts to sweat or gets out of breath

Cohen’s d*: if higher scores of the outcome parameter describe worse health condition (e.g. GCPS-parameters), minus d means IG is superior to CG and plus d means IG is worse than CG; if higher scores describe better health conditions (e.g. SF-12 parameters), minus d signifies IG is inferior to CG and plus d means IG is better than CG

In comparison to the CG, the participants of the IG presented themselves at the 2-year follow-up with less BP-dependent disability and demonstrated improved scores in their physical health status (SF-12). There were no significant differences between the groups at the 2-year follow-up in intensity of BP and mental health.

Both the psychological distress (total score of the PHQ-4) and the risk of BP chronification (total score of the STarT-G) were lower in the IG than in the CG. The IG reported more days per week with at least 10 min of physical activity than the CG.

All observed significant differences in the patient-reported outcomes between IG and CG correspond to small effect sizes (range of d: 0.21–0.26).

### Ancillary analyses

In addition to the main analysis, treatment effects in the primary outcomes were separately analyzed in two subgroups consisting of study participants with either low risk of BP chronification (STarT-G total score not exceeding 3) or with medium or high risk (STarT-G total score greater than 3) at baseline.

Significant long-term effects only occurred in the subgroup with medium or high risk of BP chronification (Table 5). In this subgroup, intensity of BP and disability (GCPS) were lower and the physical health status (SF-12) was higher in the IG than in CG with effect sizes of approximately 0.4. Only the difference in mental health status did not reach significance.

**Table 5:** Subgroup analyses: treatment effects within two STarT Back risk groups

Primary outcomes		STarT-G score at baseline (0–3) (low risk of back pain chronification)				STarT-G score at baseline > 3 (= medium or high risk of back pain chronification)			
		IG (*N* = 100) M ^+^ (95% CI)	CG (*N* = 162) M ^+^ (95% CI)	Between group effects		IG (*N* = 89) M ^+^ (95% CI)	CG (*N* = 93) M ^+^ (95% CI)	Between group effects	
				p	Cohen’s d * [95% CI]			p	Cohen’s d * [95% CI]
Back pain severity (CPGQ)	pain intensity (0–100)	33.3 (29.9–36.7)	32.3 (29.6–34.9)	0.632	0.06 (−0.19 to 0.31)	47.5 (43.8–51.3)	54.9 (51.2–58.6)	0.007	−0.41(− 0.70 to − 0.11)
	disability points (0–6)	1.1 (0.8–1.4)	1.1 (0.9–1.4)	0.778	0 (− 0.25 to 0.25)	2.5 (2.1–2.9)	3.2 (2.8–3.5)	0.018	− 0.36(− 0.65 to − 0.06)
Health- related quality of life (SF-12)	physical component (0–100)	46.3 (44.6–48.1)	45.4 (44.1–46.8)	0.416	0.10 (− 0.15 to 0.35)	38.5 (36.6–40.3)	34.6 (32.8–36.5)	0.005	0.43 (0.13 to 0.72)
	mental component (0–100)	48.0 (46.2–49.9)	50.1 (48.6–51.5)	0.090	−0.22 (− 0.47 to 0.03)	44.7 (42.4–46.9)	41.9 (39.7–44.1)	0.094	0.25 (− 0.04 to 0.55)

*IG* intervention group, *CG* control group, *p* significance value, M ^+^: values of the group means adjusted for baseline-score, StarT-G total risk score and satisfaction with medical care of back pain at baseline, *CI* Confidence interval, CPGQ Chronic Pain Grade Questionnaire; SF-12: Short From 12

Cohen’s d*: if higher scores of the outcome parameter describe worse health condition (GCPS-parameters), minus d means IG is superior to CG and plus d means IG is worse than CG; if higher scores describe better health conditions (SF-12 parameters), minus d signifies IG is inferior to CG and plus d means IG is better than CG

Altman’s between-subgroup interaction test was used to examine whether this heterogeneity in treatment effects depends on the person’s risk-level of BP chronification at baseline (see Table 6).

**Table 6:** Subgroup analyses: differences in treatment effects between subgroups (statistical test of interaction)

Primary outcomes			Subgroups	
			Low risk (0–3) IG versus CG	Medium/High risk (> 3) IG versus CG
Back pain severity (CPGQ)	characteristic back pain intensity (0–100)	Mean Difference (SE) between IG / CG	- 1.0 (2.17)	+ 7.4 (2.69)
		Δ of treatment effect in the both subgroups (SE)	8.4 (3.46)	
		z-score/ p	2.428/ 0.015	
	disability points (0–6)	mean difference (SE) between IG / CG	0.05 (0.22)	0.68 (0.28)
		Δ of treatment effect in the both subgroups (SE)	0.63 (0.36)	
		z-score/ p	1.750 / 0.080	
Health-related quality of life (SF-12)	physical component (0–100)	mean difference (SE) between IG / CG	0.9 (1.10)	3.9 (1.34)
		Δ of treatment effect in the both subgroups (SE)	3.0 (1.73)	
		z-score/ p	1.734 / 0.083	
	mental component (0–100)	mean difference (SE) between IG / CG	- 2.1 (1.38)	2.7 (1.60)
		Δ of treatment effect in the both subgroups (SE)	4.8 (2.11)	
		z-score/ p	2.275/ 0.023	

*CPGQ* Chronic Pain Grade Questionnaire according to v. Korff, *SF-12* 12 Items Short Form questionnaire, *SE* standard error, Δ: difference, *p* significance value

The results of the interaction tests suggest that persons scoring higher in STarT Back Screening Tool at baseline benefit significantly more from the health program than persons with low risk scores.

## Discussion

A German private medical insurance proactively offered selected members with chronic BP a health program that included multidisciplinary treatment for up to 1 year. Feasibility and efficacy of this approach were evaluated by a randomized controlled trial using Zelen’s design. The results of the 2-year follow-up favor the chosen approach. The proactive approach of the health insurance company in offering BP program to selected insured persons with chronic BP proved to be a feasible way of recruiting participants to a scientific study evaluating the effects of such a program. The recruiting strategy proved successful in identifying the appropriate target groups. The study participants had BP of similar severity (44% with chronic pain grades III or IV) such as BP patients seen at German family practices (45% with chronic pain grade III or IV [24]. They were more severely impaired than a German population cohort (11% with chronic pain grade III or IV, [39] and less impaired than patients with BP treated in pain clinics (85% with chronic pain grade III or IV, [40].

A year after the end of the program, members of the IG reported significantly less disability and had better scores on the somatic HRQoL than the CG members. IG members showed less psychological distress, had a smaller risk of BP chronification and were also more physically active than the CG members. There were no differences between the two groups in pain intensity and mental HRQoL.

Subgroup analyses showed that especially study participants with medium or high risk of chronification at baseline (STarT-G score > 3) benefit from the intervention whereas no differences between IG and CG were seen in the low-risk group in BP severity and HRQoL.

All the observed significant long-term effects were on average small, but these results are promising in the light of the existing literature. In a recently published review [41] including data of 41 trials assessing the long-term effects of multidisciplinary rehabilitation interventions for chronic BP, it was reported that such interventions were more effective than usual care in decreasing pain and disability, with small effect sizes. Other reviews have reported comparable small long-term effects [42–44].

The question arises if such small effects are clinically relevant. Estimating a minimum clinically important difference (MCID) has been a challenging subject since three decades. Different methodologies (anchor-based, distribution-based) for determining MCID are used and the optimal method has remained controversial (see [45–47]). For estimating the clinical relevance of at least one of the observed significant small effects, we defined according to [48], an MCID of 3.29 points for the physical component scale of the SF-12. With this approach, relevant improvements were found more frequently in the IG than in the CG (52.4% vs 40.8%; *p* = 0.015).

### Strengths of the study

Although health care policy requires scientifically sound evaluation of health care innovations, unproven innovations are too often implemented in health care systems. Since 2016, German statutory health insurances can apply for funds by the newly created Innovation Fund (worth € 300 million per year) for health services-related research projects. However, private health insurances have no access to this fund. It is to the credit of Central as a private health insurance company that they made an effort to get their new health program evaluated and its efficacy examined not in the short term - where effects are generally larger - but in the long term.

### Limitations of the study

Since a conventional RCT design (randomization after informed consent) carries with it a risk of dissatisfaction on the part of the members of the non-preferred arm, a “post randomization consent design” according to Zelen was chosen, which, however, is not uncontroversial [49–51]. Different participation rates in IG and CG and numerous baseline differences between IG and CG are regarded as typical disadvantages of using such a design. Both occurred in our evaluation study reducing the comparability of the study arms. The invitation of the health insurance company to participate in the health program with accompanying evaluation (IG) was accepted by chance by fewer insured persons than the invitation to participate in a long-term observation of their BP problems (CG). The difference in the willingness to participate in the study is probably due to the significantly different time and personal commitment required from study subjects. Participation in the CG was limited to filling out an online questionnaire several times, while participation in the IG was associated with a variety of requirements (including visits to the doctor, muscle training, telephone calls from the coach).

As is frequently the case in health services research, our study participants could not be blinded to the treatment they received. The only thing they were not told was about the randomized group allocation based on the Zelen’s design we used in our study. The physicians administering the interventions to the IG and those taking care of the CG were not aware of the evaluation study.

Furthermore, only patient-reported variables were used as study outcomes. However, taking into account the absence of any dependency of the participants on the researcher handling the data, the risk of social desirability bias can be assumed to be low.

The influence of possible moderators and mediators such as comorbidity or operations on the outcomes could not be evaluated because such data were not available.

The interesting question of whether sociodemographic variables (such as age, gender, formal education) were (or were not) associated with treatment outcomes remains unanswered, being outside the scope of the study.

A sample of members of a single private health insurance does not provide a representative picture of the German population. As is known [52, 53], members of German private health insurances (about 15% of the German population) differ in sociodemographic and health-related characteristics from members covered by statutory health insurances. For instance, they have better than average levels of education. The study results are, therefore, not generalizable.

An attrition bias cannot be excluded. We considered only complete cases and it might be that persons lost to follow-up at 24 months had better or worse outcomes resulting in an under- or overestimation of true effects. At the 12-month follow-up the drop-out rate was high. However, with reorganization of follow-up management, it was possible to reduce the lost-to-follow-up rates and thus overcome the threshold of 30% set for judgment of risk of attrition bias (see [54]). Hence, any attrition bias in this study was likely not substantial.

Additionally, the possibility that analyses of multiple primary and secondary outcomes could have increased the risk of significant effects by chance (i.e. inflation of α-error) cannot be excluded.

### Outlook

We identified two possible points of improvement for the future use of the health program. On the one hand, before inviting patients to participate in the program, it is necessary to ascertain the extent to which network doctors and associated therapy centers can be found within easy reach of the insured person’s place of residence. Ensuring easy access in terms of distance might increase program acceptance and adherence. Furthermore, the health program offered should not be based on an “one-size fits all” concept. The positive effects might be increased by the use of the STarT back tool to stratify eligible participants with BP into low, medium and high risk of BP chronification with special care pathways for the three subgroups. The predictive and discriminative ability of the STarT back tool in populations with BP of variable episode duration is widely supported in the literature (inter alia [55–58]). Sophisticated treatment systematically targeting medium and high-risk groups apparently leads to improved outcomes [59]. Our results suggest that the low-risk subgroup derives hardly any benefits from the health program in the long-term; with screening, potential overtreatment of the low-risk subgroup probably needing only minimal treatment can be avoided.

In summary, the available results of the present study support continuing the program. Approaches for increasing the observed beneficial effects have been mentioned above. An analysis of the cost data is pending, so that a final cost-benefit assessment has not yet been carried out.

## Conclusions

The results of the study strengthen the assumption that it is feasible and beneficial to address persons at risk for chronic diseases (e.g. chronic BP) directly through their health insurances and invite them to utilize evidence-based care.

The proactive health program “initiative.back” proved to be effective and beneficial in improving the relevant long-term patient-reported outcomes such as BP-related disability and physical HRQoL to a greater extent than usual care. In the future, the observed positive effects could be strengthened by using a screening tool like the STarT back tool to offer the program only to persons with medium or high risk of poor prognosis. Acceptance of the health program can be enhanced by therapy centers that are within easy reach of the patient’s place of residence.

## Data Availability

The datasets analyzed during the current study will be shared with researchers who provide a methodologically sound proposal. Proposals should be directed to the corresponding author. To gain access data requestors need to sign a data access agreement.

## Abbreviations

ANCOVA: Analysis of Covariance
BP: Back Pain
CG: Control Group
CPGQ: Chronic Pain Grade Questionnaire
DMP: Disease Management Program
FPZ: Forschungs- und Präventionszentrum (research- and prevention- centre)
HRQoL: Health-related Quality of Life
IG: Intervention Group
NRS: Numerical Rating Scale
PHQ-4: Patient Health Questionnaire-4
SF-12: 12-Item Short Form Health Survey
STarT-G: Keele STarT Back Tool, German version
vs: versus

## References

[1] Raspe H (2012). [Back pain] [German]. Federal Health Reporting Booklet 53.

[2] Plass D, Vos T, Hornberg C, Scheidt-Nave C, Zeeb H, Kramer A (2014). Trends in disease burden in Germany: results, implications and limitations of the global burden of disease study. Dtsch Arztebl Int.

[3] Hoy D, March L, Brooks P, Blyth F, Woolf A, Bain C (2014). The global burden of low back pain: estimates from the global burden of disease 2010 study. Ann Rheum Dis.

[4] Grobe T (2014). Risiko Rücken. Gesundheitsreport 2014.

[5] Wenig CM, Schmidt CO, Kohlmann T, Schweikert B (2009). Costs of back pain in Germany. Eur J Pain.

[6] Oliveira CB, Maher CG, Pinto RZ, Traeger AC, Lin CC, Chenot JF, van Tulder M, Koes BW (2018). Clinical practice guidelines for the management of non-specific low back pain in primary care: an updated overview. Eur Spine J.

[7] van Tulder M, Becker A, Bekkering T, Breen A, del Real MT, Hutchinson A (2006). Chapter 3. European guidelines for the management of acute nonspecific low back pain in primary care. Eur Spine J.

[8] Chenot J-F, Greitemann B, Kladny B, Petzke F, Pfingsten M, Schorr SG (2017). Clinical practice guideline. Non-specific low Back pain. Dtsch Arztebl Int.

[9] Bundesärztekammer (BÄK), Kassenärztliche Bundesvereinigung (KBV), Arbeitsgemeinschaft der Wissenschaftlichen Medizinischen Fachgesellschaften (AWMF). [National Disease Management Guideline Non Specific Low Back Pain – Long Version] [German] 2nd edition. Version 1. https://www.leitlinien.de/mdb/downloads/nvl/kreuzschmerz/kreuzschmerz-2aufl-vers1-lang.pdf Accessed 2 May 2019.

[10] Slade SC, Kent P, Patel S, Bucknall T, Buchbinder R (2016). Barriers to primary care clinician adherence to clinical guidelines for the Management of low Back Pain: a systematic review and Metasynthesis of qualitative studies. Clin J Pain.

[11] Werber A, Schiltenwolf M (2016). Treatment of lower Back pain-the gap between guideline-based treatment and medical care reality. Healthcare.

[12] Marstedt G (2016). Faktencheck Rücken: Einstellungen, Erfahrungen, Informationsverhalten – Bevölkerungsumfrage zum Rückenschmerz; Bertelsmann Stiftung.

[13] Darlow B, Fullen BM, Dean S, Hurley DA, Baxter GD, Dowell A (2012). The association between health care professional attitudes and beliefs and the attitudes and beliefs, clinical management, and outcomes of patients with low back pain: a systematic review. Eur J Pain.

[14] Institute for Quality and Efficiency in Health Care (IQWIG) (2015). Systematic Guideline Search and Appraisal, as Well as Extraction of Relevant Recommendations, for a DMP “Chronic Back Pain”. Cologne.

[15] Marnitz U, Weh L, Muller G, Seidel W, Bienek K, Lindena G (2008). Multimodal integrated assessment and treatment of patients with back pain. Pain related results and ability to work [German]. Schmerz.

[16] Lindena G, Marnitz U, Hartmann P, Müller G (2012). “Back pain coach”. A project for patients with back pain [German]. Schmerz.

[17] Hüppe Angelika, Wunderlich Max, Hochheim Martin, Mirbach Andrea, Zeuner Christel, Raspe Heiner (2017). Evaluation eines proaktiv angebotenen Gesundheitsprogramms für Versicherte mit anhaltenden Rückenschmerzen – Ein-Jahres-Follow-up einer randomisierten, kontrollierten Studie. Das Gesundheitswesen.

[18] Zelen M (1990). Randomized consent designs for clinical trials: an update. Stat Med.

[19] Flory JH, Mushlin AI, Goodman ZI (2016). Proposals to conduct randomized controlled trials without informed consent: a narrative review. J Gen Intern Med.

[20] Freytag A, Schiffhorst G, Thoma R, Strick K, Gries C, Becker A (2010). [Identification and grouping of pain patients according to claims data] [German]. Schmerz.

[21] Schiffhorst G, Freytag A, Höer A, Häussler B, Gothe H (2010). Pain-specific diagnosis patterns in claims data – identification by means of classification and regression trees (CART) [German]. Das Gesundheitswesen.

[22] Denner A (1998). Analyse und Training der wirbelsäulenstabilisierenden Muskulatur.

[23] Von Korff M, Ormel J, Keefe FJ, Dworkin SF (1992). Grading the severity of chronic pain. Pain.

[24] Klasen BW, Hallner D, Schaub C, Willburger R, Hasenbring M (2004). Validation and reliability of the German version of the Chronic Pain Grade questionnaire in primary care back pain patients. Psychol Med.

[25] Morfeld M, Kirchberger I, Bullinger M (2011). SF-36 Fragebogen zum Gesundheitszustand: Deutsche Version des Short Form-36 Health Survey.

[26] Karstens S, Krug K, Hill JC, Stock C, Steinhaeuser J, Szecsenyi J (2015). Validation of the German version of the STarT-Back tool (STarT-G): a cohort study with patients from primary care practices. BMC Musculoskelet Disord.

[27] Karstens S, Krug K, Raspe H, Wunderlich M, Hochheim M, Joos S (2019). Prognostic ability of the German version of the STarT Back tool: analysis of 12-month follow-up data from a randomized controlled trial. BMC Musculoskelet Disord.

[28] Hill JC, Dunn KM, Lewis M, Mullis R, Main CJ, Foster NE (2008). A primary care back pain screening tool: identifying patient subgroups for initial treatment. Arthritis Rheum.

[29] Kroenke K, Spitzer RL, Williams JBW, Löwe B (2009). (2009). An ultra-brief screening scale for anxiety and depression: the PHQ-4. Psychosomatics.

[30] Löwe B, Wahl I, Rose M, Spitzer C, Glaesmer H, Wingenfeld K (2010). A 4-item measure of depression and anxiety: validation and standardization of the patient health Questionnaire-4 (PHQ-4) in the general population. J Affect Disord.

[31] Krug S, Jordan S, Mensink GB, Muters S, Finger J, Lampert T (2013). [Physical activity: results of the German health interview and examination survey for adults (DEGS1)] [German]. Bundesgesundheitsbl Gesundheitsforsch Gesundheitsschutz.

[32] Abt K (1987). Descriptive data analysis: a concept between confirmatory and exploratory data analysis. Methods Inf Med.

[33] Hedges L, Olkin I (1985). Statistical methods for meta-analysis.

[34] Altman DG, Matthews JNS (1996). Statistics notes: interaction 1: heterogeneity of effects. BMJ.

[35] Matthews JN, Altman DG (1996). Statistics notes. Interaction 2: Compare effect sizes not P values. BMJ.

[36] Matthews JN, Altman DG (1996). Interaction 3: how to examine heterogeneity. BMJ.

[37] Altman DG, Bland JM (2003). Interaction revisited: the difference between two estimates. BMJ.

[38] Lenhard W, Lenhard A (2016). Calculation of Effect Sizes.

[39] Schmidt CO, Raspe H, Pfingsten M, Hasenbring M, Basler HD, Eich W (1976). Back pain in the German adult population: prevalence, severity, and sociodemographic correlates in a multiregional survey. Spine.

[40] Nagel B, Pfingsten M, Lindena G (2012). Handbuch Deutscher Schmerzfragebogen. Revision 2012.2.

[41] Kamper SJ, Apeldoorn AT, Chiarotto A, Smeets RJ, Ostelo RW, Guzman J (2014). Multidisciplinary biopsychosocial rehabilitation for chronic low back pain. Cochrane Database Syst Rev.

[42] Hüppe A, Raspe H (2005). [Efficacy of inpatient rehabilitation for chronic back pain in Germany: update of a systematic review] [German]. Rehabilitation.

[43] van Middelkoop M, Rubinstein SM, Kuijpers T, Verhagen AP, Ostelo R, Koes BW (2011). A systematic review on the effectiveness of physical and rehabilitation interventions for chronic non-specific low back pain. Eur Spine J.

[44] van Middelkoop M, Rubinstein SM, Verhagen AP, Ostelo RW, Koes BW, van Tulder MW (2010). Exercise therapy for chronic nonspecific low-back pain. Best Pract Res Clin Rheumatol.

[45] Jaeschke R, Singer J, Guyatt GH (1989). Measurement of health status. Ascertaining the minimal clinically important difference. Control Clin Trials.

[46] Gatchel RJ, Lurie JD, Mayer TG (2010). Minimal clinically important difference. Spine.

[47] Angst F, Aeschlimann A, Angst J (2017). The minimal clinically important difference raised the significance of outcome effects above the statistical level, with methodological implications for future studies. J Clin Epidemiol.

[48] Diaz-Arribas MJ, Fernandez-Serrano M, Royuela A, Kovacs FM, Gallego-Izquierdo T, Ramos-Sanchez M (2017). Minimal clinically important difference in quality of life for patients with low Back pain. Spine.

[49] Torgerson DJ, Roland M (1998). What is Zelen's design?. BMJ.

[50] Fan AY (2015). The methodology flaws in Hinman's acupuncture clinical trial, part II: Zelen design and effectiveness dilutions. J Integr Med.

[51] Homer CS (2002). Using the Zelen design in randomized controlled trials: debates and controversies. J Adv Nurs.

[52] Hoffmann F, Koller D (2017). [Different Regions, Differently Insured Populations? Socio-demographic and Health-related Differences Between Insurance Funds] [German]. Gesundheitswesen.

[53] Stauder J, Kossow T (2017). [Selection or Better Service - Why are those with Private Health Insurance Healthier than those Covered by the Public Insurance System?] [German]. Gesundheitswesen.

[54] Furlan AD, Malmivaara A, Chou R, Maher CG, Deyo RA, Schoene M (2015). 2015 updated method guideline for systematic reviews in the Cochrane Back and neck group. Spine.

[55] Morso L, Kent P, Manniche C, Albert HB (2014). The predictive ability of the STarT Back screening tool in a Danish secondary care setting. Eur Spine J.

[56] Page I, Abboud J (2015). J OS, Laurencelle L, Descarreaux M. chronic low Back pain clinical outcomes present higher associations with the STarT Back screening tool than with physiologic measures: a 12-month cohort study. BMC Musculoskelet Disord.

[57] Kendell M, Beales D, O'Sullivan P, Rabey M, Hill J, Smith A (2018). The predictive ability of the STarT Back tool was limited in people with chronic low back pain: a prospective cohort study. J Phys.

[58] Suri P, Delaney K, Rundell SD, Cherkin DC (2018). Predictive validity of the STarT Back tool for risk of persistent disabling Back pain in a U.S. primary care setting. Arch Phys Med Rehabil.

[59] Meyer C, Denis CM, Berquin AD (2018). Secondary prevention of chronic musculoskeletal pain: a systematic review of clinical trials. Ann Phys Rehabil Med.

